# Pleomorphic Adenoma of the Soft Palate With Indeterminate Imaging and Nondiagnostic Fine-Needle Aspiration Cytology: A Case Report

**DOI:** 10.7759/cureus.116536

**Published:** 2026-09-19

**Authors:** Afrah Fathima Karimbanakkal Edakkattu, Abdul Salam RT

**Affiliations:** 1 Medicine, Government Medical College Manjeri, Kerala University of Health Sciences, Manjeri, IND; 2 Ear, Nose, and Throat (ENT), Government Medical College Manjeri, Kerala University of Health Sciences, Manjeri, IND

**Keywords:** fine-needle aspiration cytology (fnac), minor salivary glands, palatal swelling, pleomorphic adenoma, salivary gland neoplasm, soft palate

## Abstract

Pleomorphic adenoma is the most common benign salivary gland neoplasm and may arise from the minor salivary glands of the palate. Palatal masses can resemble inflammatory, infectious, or other neoplastic lesions, and fine-needle aspiration cytology (FNAC) may be nondiagnostic. A 35-year-old woman presented with a painless, gradually enlarging swelling of the left soft palate of three months' duration. Examination revealed a smooth, firm, non-tender, non-ulcerated submucosal mass measuring approximately 3 × 2 cm and extending toward the left tonsillar fossa. Contrast-enhanced computed tomography demonstrated a well-circumscribed heterogeneous lesion measuring 2.3 × 2.3 × 2 cm adjacent to the left tonsillar fossa, with peripheral enhancement, a hypoenhancing center, and mass effect on the soft palate and pharyngeal mucosa. An inflammatory or phlegmonous process was considered, although neoplasia could not be excluded. FNAC yielded predominantly hemorrhagic material with scattered plasmacytoid cells and occasional cell clusters and was insufficient for a definitive diagnosis. The lesion was excised in toto through a transoral approach under general anesthesia, and its capsule was grossly intact. Gross examination revealed a well-circumscribed, firm, gray-white nodular specimen measuring 2 × 2 × 2 cm. Histopathological examination confirmed pleomorphic adenoma. No local recurrence was documented at the six-month follow-up. This case emphasizes that salivary gland neoplasia should remain in the differential diagnosis of persistent palatal masses despite inflammatory-appearing imaging and nondiagnostic FNAC, with histopathological examination providing the definitive diagnosis.

## Introduction

Pleomorphic adenoma, also known as benign mixed tumor, is the most common benign salivary gland neoplasm. It may arise in major or minor salivary glands and is characterized by variable epithelial and myoepithelial differentiation within a heterogeneous stromal background. In a demographic and histopathological study of 426 intraoral minor salivary gland tumors, the palate was the most frequent anatomical site, and pleomorphic adenoma was the most common benign tumor [1].

Pleomorphic adenomas of minor salivary glands usually present as slow-growing, painless, firm, well-circumscribed submucosal masses with preserved overlying mucosa. However, palatal swelling has a broad differential diagnosis that includes infectious and inflammatory lesions, odontogenic lesions, vascular lesions, benign salivary gland tumors, and malignant neoplasms [1,2].

Evaluation generally combines clinical examination, imaging, and tissue sampling. Computed tomography defines lesion size, location, local extension, and relationships with adjacent structures, but heterogeneous enhancement or central low attenuation may not be pathognomonic [2]. Fine-needle aspiration cytology (FNAC) is useful in the assessment of salivary gland lesions, although inadequate cellularity, selective sampling, lesion heterogeneity, and overlapping cytological features may limit its diagnostic value [3,4].

Soft-palate pleomorphic adenoma has been described in both small localized lesions and large tumors requiring extensive surgical access [5,6]. This case illustrates a diagnostic challenge in which imaging was indeterminate and FNAC was nondiagnostic; the definitive diagnosis was established only after surgical excision and histopathological examination.

## Case presentation

Patient information

A 35-year-old woman presented to the otorhinolaryngology outpatient department of Government Medical College Manjeri, Kerala, India, with a left soft-palate swelling that had been present for approximately three months. She had noticed the lesion incidentally and reported gradual enlargement. The swelling was painless and was not associated with dysphagia, foreign-body sensation, voice change, aspiration, nasal regurgitation, earache, neck swelling, fever, or weight loss. There was no history of local trauma, relevant chronic illness, or substance use.

The clinical course is summarized in Table 1.

**Table 1:** Clinical timeline of presentation, diagnostic evaluation, treatment, and follow-up. FNAC: fine-needle aspiration cytology

Date or period	Clinical event
Day 1	The patient noticed a small swelling over the left side of the soft palate.
Day 55	Contrast-enhanced computed tomography of the neck demonstrated a 2.3 × 2.3 × 2 cm heterogeneous lesion adjacent to the left tonsillar fossa.
Day 76	FNAC yielded predominantly blood with scattered plasmacytoid cells and occasional cell clusters; no definitive diagnosis was established.
Day 84	Transoral excision in toto of the left soft-palate lesion was performed under general anesthesia.
Day 90	Histopathological examination established pleomorphic adenoma of the soft palate.
Day 264	Six-month follow-up showed no evidence of local recurrence.

Clinical findings

Oropharyngeal examination showed an approximately 3 × 2 cm swelling involving the left soft palate. The lesion had a smooth surface and regular borders, without ulceration or necrosis. The overlying mucosa appeared normal, although the adjacent posterior mucosa was erythematous. The superior margin was approximately 1 cm below the hard-soft palate junction, and the inferior margin was approximately 1 cm above the free edge of the soft palate. The uvula was not involved, and the lesion extended toward the left tonsillar fossa. On palpation, it was firm, non-tender, clinically non-mobile, non-fluctuant, non-compressible, and non-pulsatile. No cervical lymphadenopathy was documented, and indirect laryngoscopy was unremarkable (Figure 1).

**Figure 1:**
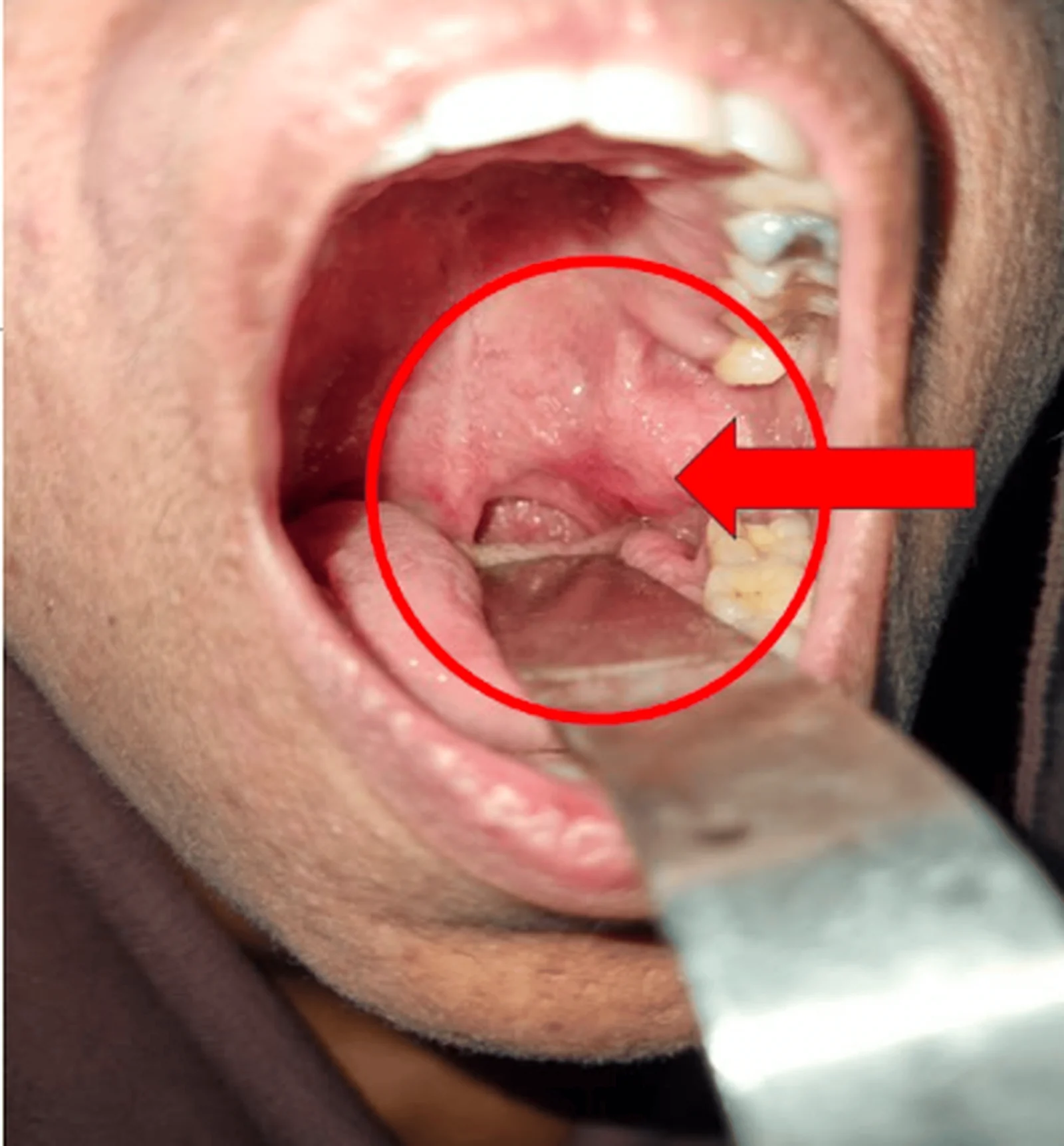
Preoperative left soft-palate mass. Intraoral examination shows a smooth, well-circumscribed submucosal swelling of the left soft palate (red arrow and circle).

The lesion extended superiorly to approximately 1 cm below the hard-soft palate junction and inferiorly to within 1 cm of the free edge of the soft palate, without the involvement of the uvula. It extended laterally toward the left tonsillar fossa and approached the level of the third molar, while medially, it was located approximately 2 cm from the midline. On palpation, the swelling was firm, non-tender, and non-mobile, with no evidence of fluctuation, compressibility, or pulsatility. The uvula, tonsils, tonsillar pillars, and posterior pharyngeal wall were otherwise unremarkable, and no cervical lymphadenopathy was noted (Figure 1).

Diagnostic assessment

Routine hematological, biochemical, and coagulation investigations were within the institution's reference ranges. Electrocardiography showed normal sinus rhythm, and chest radiography was reported as within normal limits.

Contrast-enhanced computed tomography

Contrast-enhanced computed tomography of the neck demonstrated a well-circumscribed oval heterogeneous lesion measuring approximately 2.3 × 2.3 × 2 cm adjacent to the left tonsillar fossa. It showed heterogeneous enhancement, with an enhancing peripheral component and a hypoenhancing center. The lesion produced anterior mass effect on the soft palate and lateral mass effect on the pharyngeal mucosa. The fat plane with the left medial pterygoid muscle was preserved (Figures 2, 3). The appearance was indeterminate; an infective or phlegmonous process involving the left tonsillar or peritonsillar region was considered, although an underlying neoplasm could not be excluded.

**Figure 2:**
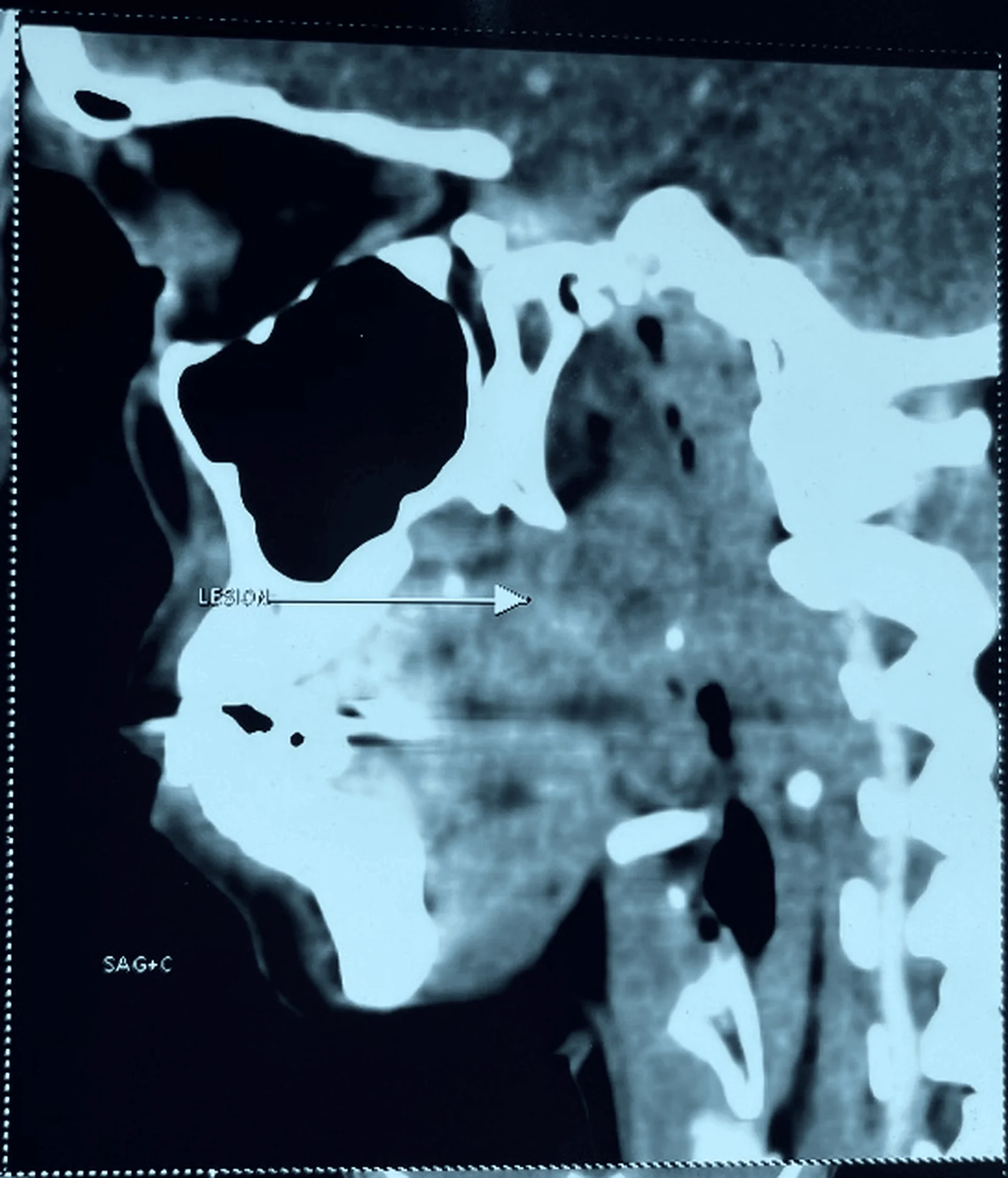
Sagittal contrast-enhanced computed tomography. The sagittal image shows a well-circumscribed heterogeneous lesion adjacent to the left tonsillar fossa with peripheral enhancement and central hypoenhancement (white arrow).

**Figure 3:**
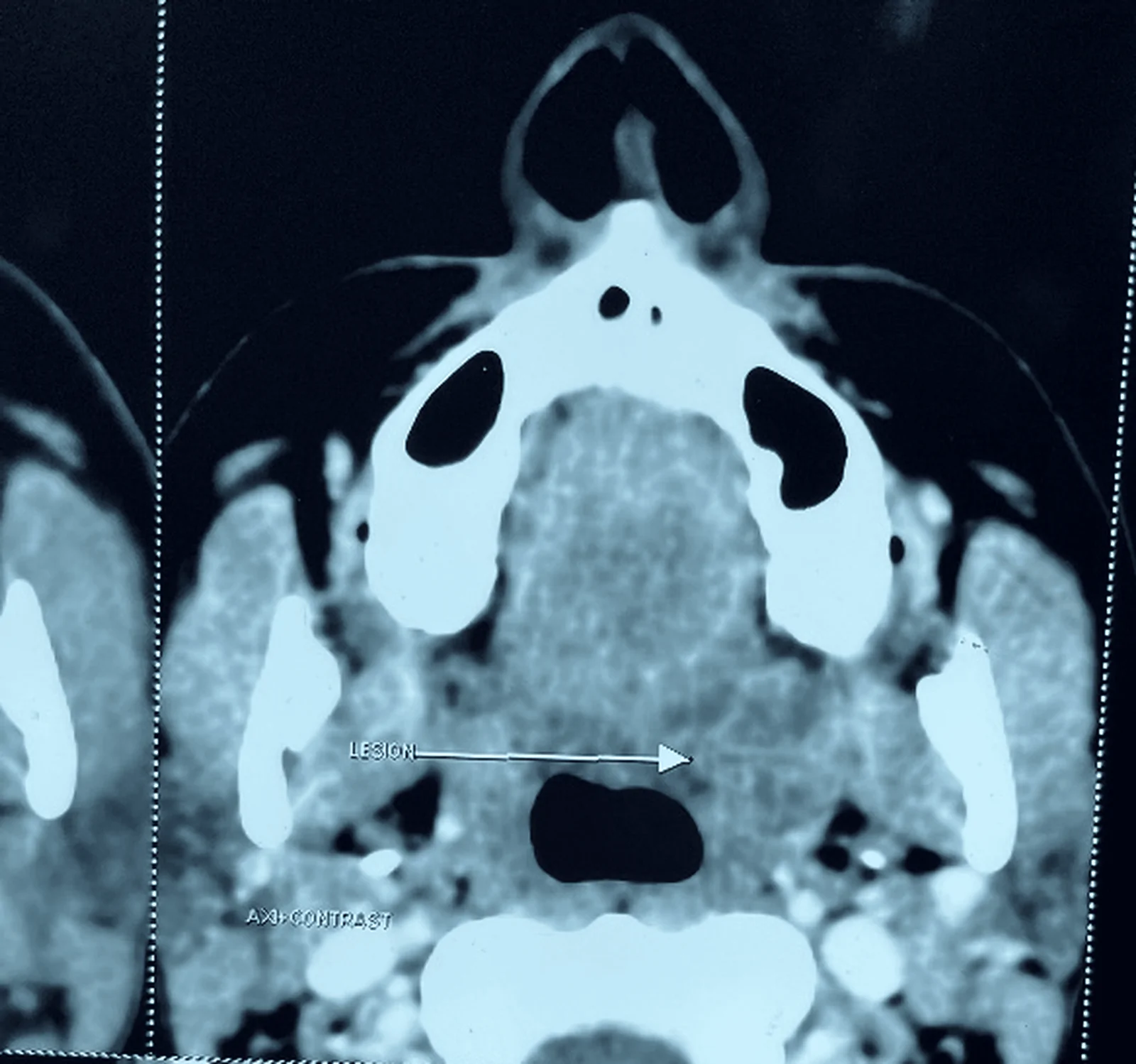
Axial contrast-enhanced computed tomography. The axial image shows the left palatal and peritonsillar lesion with associated mass effect (white arrow).

Fine-needle aspiration cytology

FNAC yielded predominantly blood with scattered plasmacytoid cells and occasional cell clusters. The specimen was insufficient for a definitive diagnosis, and clinical and radiological correlation was recommended. Plasmacytoid cells may represent the myoepithelial component of pleomorphic adenoma, but the limited cellularity and blood-dominant aspirate prevented definitive classification. This finding is consistent with recognized limitations of FNAC in salivary gland lesions [3,4].

Differential diagnosis

The principal differential diagnoses were pleomorphic adenoma and other minor salivary gland neoplasms, including mucoepidermoid carcinoma, adenoid cystic carcinoma, and polymorphous adenocarcinoma. An infective or phlegmonous lesion of the tonsillar or peritonsillar region was also considered because of the computed tomography appearance. Persistence, painless progression, firm consistency, preserved mucosa, and the absence of systemic or laboratory evidence of acute infection maintained concern for an underlying neoplasm.

Therapeutic intervention

Because the lesion persisted and an underlying neoplasm could not be excluded despite nondiagnostic FNAC, surgical excision was planned for diagnosis and treatment. Written informed consent for the procedure was obtained.

The patient underwent transoral excision of the left soft-palate lesion under general anesthesia. During exploration, the lesion appeared smooth, firm, mobile, well-delineated, and grossly encapsulated. After local infiltration with lignocaine and adrenaline, a mucosal incision was made using monopolar cautery, followed by blunt dissection. The lesion was excised in toto with the gross capsule intact and without apparent rupture or spillage. Hemostasis was secured, and the defect was closed primarily using 2-0 Vicryl (Ethicon, Inc., Raritan, NJ) (Figure 4). The postoperative course was uneventful.

**Figure 4:**
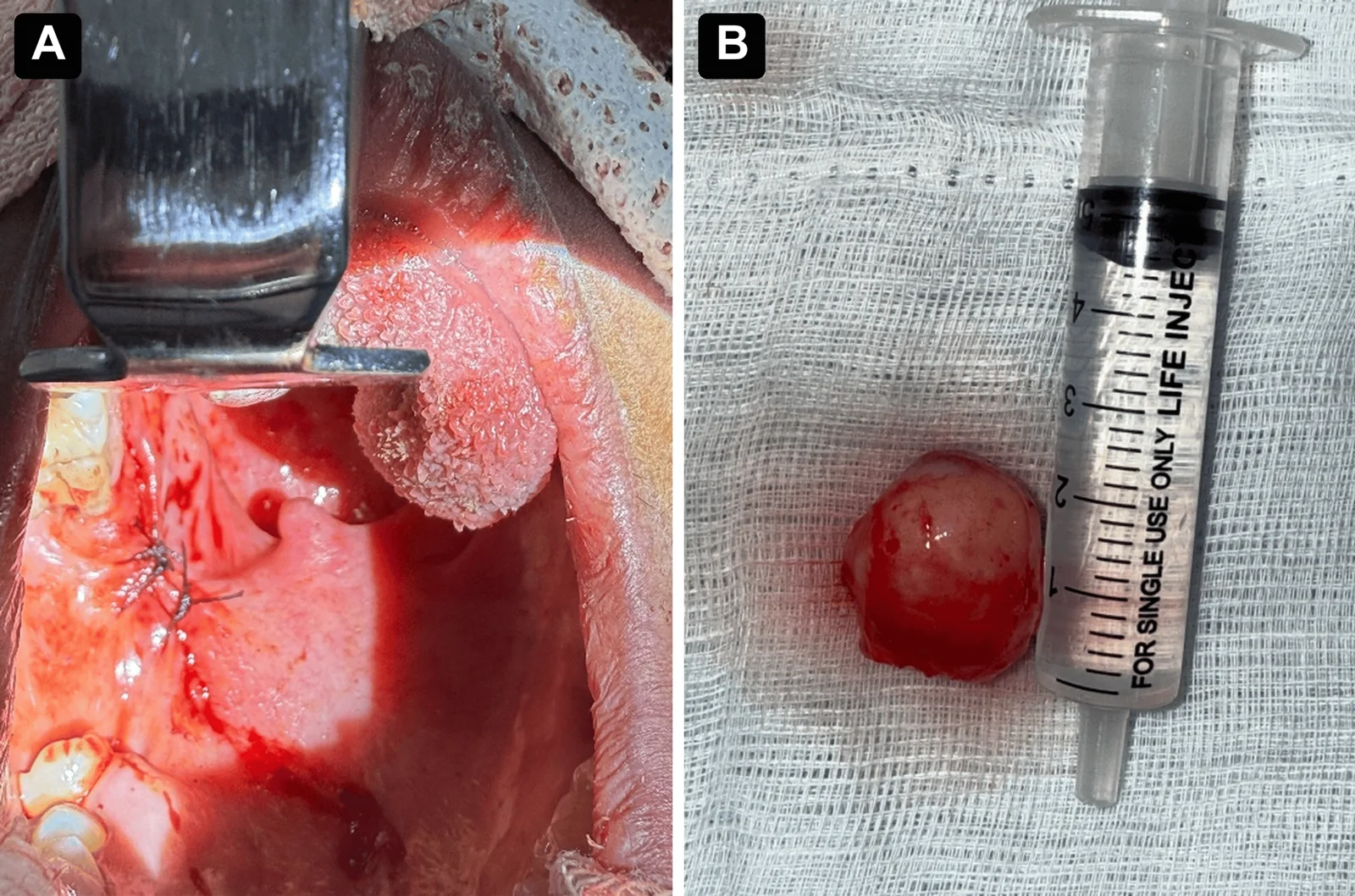
Operative site and excised specimen. (A) Surgical bed after transoral excision and primary closure. (B) The excised, well-circumscribed lesion is shown adjacent to a 5 mL syringe for scale.

Patient perspective

The patient was informed about the persistent nature of the lesion, the limitations of the initial FNAC findings, and the need for surgical excision to establish a definitive diagnosis and provide treatment. After discussing the potential benefits, risks, and alternatives, the patient provided written informed consent and underwent the procedure. The patient tolerated the surgery well and reported no significant postoperative concerns during follow-up.

Histopathological diagnosis

The histopathological examination of the excised specimen established the diagnosis of pleomorphic adenoma of the soft palate. Pleomorphic adenomas of minor salivary glands may show variable combinations of epithelial, myoepithelial, and stromal components, which account for their characteristic histological heterogeneity [7,8].

Follow-up and outcome

No local recurrence was documented at six months after surgery. This early outcome is favorable but does not establish long-term cure because recurrence may occur years after treatment. A nationwide observational study reported a 20-year recurrence rate of 6.7% for salivary gland pleomorphic adenoma, and a systematic review of recurrent palatal pleomorphic adenoma found that recurrence may be substantially delayed [9,10]. Continued clinical follow-up was therefore advised.

## Discussion

Pleomorphic adenoma may arise from minor salivary glands distributed throughout the upper aerodigestive tract, and the palate is the most frequent intraoral site. Large clinicopathological series have identified pleomorphic adenoma as a major component of benign minor salivary gland neoplasia [1,11].

The clinical presentation in this patient was typical in several respects: a painless, gradually enlarging, firm, non-ulcerated palatal swelling with preserved overlying mucosa. Comparable soft-palate pleomorphic adenomas have been managed by local excision, although larger lesions may require more extensive surgical access [5,6]. The broad differential diagnosis of palatal swelling requires both inflammatory and neoplastic causes to be considered [2].

The main diagnostic challenge was the absence of a definitive preoperative diagnosis. Computed tomography showed a well-circumscribed heterogeneous lesion with peripheral enhancement and central hypoenhancement. Although neoplasia remained possible, the radiological differential included an infective or phlegmonous process. This demonstrates that imaging can define morphology and anatomical relationships without reliably establishing histology.

FNAC added uncertainty because the aspirate was predominantly hemorrhagic and contained only scattered plasmacytoid cells and occasional cell clusters. Plasmacytoid myoepithelial cells are a recognized component of pleomorphic adenoma, but confident cytological diagnosis usually requires the characteristic combination of epithelial cells, myoepithelial cells, and stromal material. Limited cellularity, selective sampling, and morphological overlap are recognized diagnostic pitfalls [3,4].

Surgical excision resolved the diagnostic uncertainty and provided definitive treatment. Complete excision is preferred for a localized palatal pleomorphic adenoma because it provides tissue for histopathological diagnosis and reduces the likelihood of residual tumor [5,6]. In this case, the lesion was removed through a transoral approach without apparent capsular rupture.

Long-term surveillance remains important. Valstar et al. reported an overall 20-year recurrence rate of 6.7% and identified positive or uncertain margins and younger age as risk factors for recurrence [9]. The recurrence of palatal pleomorphic adenoma has been reported 16 years after initial excision, and a recent systematic review found higher odds of detecting recurrence at 10 and 20 years than at one year [10,12]. The absence of recurrence at six months is encouraging but does not eliminate the need for continued follow-up.

The strength of this case is its clearly documented diagnostic pathway: clinical assessment, cross-sectional imaging, and FNAC did not provide a definitive diagnosis, whereas excision and histopathological examination did. The principal limitations are the absence of detailed microscopic findings and margin status and the relatively short follow-up interval. These limitations prevent individualized estimates of recurrence or malignant transformation risk.

## Conclusions

Pleomorphic adenoma of the soft palate may present as a painless, slowly enlarging submucosal mass and may remain indeterminate following imaging and FNAC. In the present case, contrast-enhanced computed tomography suggested an inflammatory or phlegmonous process, while the blood-dominant FNAC aspirate was nondiagnostic and did not exclude neoplasia. Surgical excision was therefore undertaken for definitive diagnosis and treatment. This case highlights the importance of considering a salivary gland neoplasm in the differential diagnosis of a persistent palatal mass despite inflammatory-appearing imaging or inconclusive cytology. Although the patient remained clinically well without evidence of recurrence during the six-month follow-up, the histopathological margin status was not available for assessment. Therefore, longer-term clinical surveillance may be considered while acknowledging that the limited follow-up period precludes conclusions regarding late recurrence.
